# Testing Measurement Invariance of the Dark Triad Dirty Dozen in a Belgian Adult Sample

**DOI:** 10.5334/pb.1106

**Published:** 2021-12-22

**Authors:** Ann De Buck, Lieven J. R. Pauwels, Wim Hardyns, Koen Ponnet

**Affiliations:** 1 Doctoral Researcher at the research group Institute for International Research on Criminal Policy (IRCP) at the Faculty of Law and Criminology, Ghent University (Belgium), Department of Criminology, Criminal Law and Social Law, BE; 2 Professor at the research group Institute for International Research on Criminal Policy (IRCP) at the Faculty of Law and Criminology, Ghent University (Belgium), Department of Criminology, Criminal Law and Social Law, BE; 3 Professor at the research group for Media, Innovation and Communication Technologies, Department of Communication Sciences, Ghent University, BE

**Keywords:** Dark Triad Dirty Dozen, self-control, illegitimate norms, measurement invariance, adult sample

## Abstract

The Dirty Dozen (Jonason & Webster, 2010) is a frequently used concise version of the Dark Triad to measure three socially aversive personality traits: Machiavellianism, psychopathy and, narcissism. The present study has examined measurement invariance in a sample of Belgian adults. The present study aims to assess measurement invariance of the Dutch version of the Dirty Dozen measure across gender in a large city-based representative adult sample in Belgium (*N* = 1587). Multi-group first-order confirmatory factor analysis for categorical indicators was utilized. In addition, unique associations between Dirty Dozen traits, trait self-control and, acceptance of illegitimate norms were examined in a series of structural equation models. Results indicated that the internal consistency of the Dirty Dozen subscales was good for Machiavellianism (*α* = 0.80) and narcissism (*α* = 0.80), but modest for psychopathy (*α* = 0.64). The hypothesized three correlated factors model with separate factors for Machiavellianism, psychopathy and, narcissism provided a poor fit for men and women. Invariance testing across gender showed evidence for weak invariance only, indicating that the underlying latent factors are measured the same way with the same metric in the two populations. However, we were not able to establish strong measurement invariance. Observed group differences should be interpreted with caution. Furthermore, Machiavellianism and psychopathy were strongly associated with trait self-control in both men and women. Strong correlations were found between acceptance of illegitimate norms and Dirty Dozen traits, Machiavellianism and, psychopathy, but not with narcissism.

## Introduction

Previous research suggests that the concise Dirty Dozen Dark Triad measure (the 12-item Dirty Dozen; Jonason & Webster, 2010) can be used to provide reliable assessments of gender differences in Dark Triad traits (Chiorri et al., 2019; Pechorro et al., 2019). The validity and measurement invariance across gender of the Dutch-language Dirty Dozen has been examined among a sample of Belgian Dutch-speaking adolescents (Klimstra et al., 2014). However, so far, no study has examined measurement invariance in a sample of Belgian Dutch-speaking adults. In the present study, the concise Dirty Dozen measure in adults is tested by examining (1) the internal consistency, (2) measurement invariance across gender, and (3) associations with a measure of low trait self-control and a measure of acceptance of illegitimate norms.

### Dark Triad

In 2002, Paulhus and Williams introduced the Dark Triad, a multidimensional construct that has gained widespread popularity in psychology research over the last two decades. The term refers to three malevolent and socially malicious personality traits: Machiavellianism, psychopathy and, narcissism. The Dark Triad personality traits share a common core of callousness, selfishness and, manipulative tendencies (Jones & Figueredo, 2013). Although they are considered socially undesirable, the Dark Triad traits are considered non-pathological and all three can be classified within the spectrum of ‘normal’ functioning (Furnham, Richards, & Paulhus, 2013). The trait of Machiavellianism is reflected by manipulativeness, deceptive tendencies and, a cynical worldview (Bereczkei, 2018; Christie & Geis, 1970; Jones & Figueredo, 2013; Paulhus & Williams, 2002; Szijjarto & Bereczkei, 2015). Individuals who are high in Machiavellianism are characterized by callous affect, a strategic-calculating interpersonal orientation and, the tendency to exploit others (Christie & Geis, 1970; Jones & Paulhus, 2014; Vize et al., 2018). The second trait of the Dark Triad cluster, psychopathy, finds its origin in clinical literature and practice. Psychopathy is generally associated with thrill-seeking and a lack of empathy and remorse (Hare, 1985; Paulhus & Williams, 2002). Other characteristics often associated with psychopathy are grandiosity, superficial charm, irresponsibility and, recklessness (Jones & Paulhus, 2011; Miller et al., 2017; Pailing, Boon, & Egan, 2014). Since individuals scoring high on psychopathy are impulsive, they will often abandon their long-term goals for short-term rewards. It is mainly this association with impulsivity that distinguishes psychopathy from Machiavellianism (Jones & Paulhus, 2014). As Paulhus and Williams (2002) reported in their Dark Triad theory, those with subclinical psychopathic characteristics show similar characteristics to diagnosed psychopaths, albeit to a lesser extent. The last dimension of the Dark Triad, narcissism, is generally considered the least socially aversive trait of the cluster (Pailing et al., 2014). As in the case of psychopathy, the conceptualization of narcissism is drawn from clinical literature. Narcissism is a multidimensional concept that is generally associated with a sense of grandiosity, superiority and, entitlement (van Geel et al., 2017). The concept includes multiple facets such as a vulnerable and grandiose dimension (Miller et al., 2011; Pailing et al., 2014). The grandiose dimension of narcissism is characterized by arrogance, exhibitionism, selfishness and, feelings of entitlement. The vulnerable dimension manifests as a lack of self-confidence, a need for attention or recognition and, hypersensitivity to the opinion of others (Dickinson & Pincus, 2003; Miller et al., 2012). According to Jones and Paulhus (2014), it is the grandiose variant of narcissism that is represented in the Dark Triad.

### Measurement of the Dark Triad Dirty Dozen

To measure the Dark Triad traits, research within psychology has mostly relied on three independent self-report instruments. The 20-item Mach-IV (Christie & Geis, 1970) is the most widely employed scale for assessing Machiavellianism, the 40-item Narcissistic Personality Inventory (NPI; Raskin & Terry, 1988) is commonly used for measuring narcissism, and the 64-item Self-Report of Psychopathy Scale (SRP; Hare, 1985) is frequently administered for tapping the trait of psychopathy. While there is good evidence for the reliability and validity of these scales, the impractical length of these measures (124 items in total) has limited their use because their combined administration for assessing the full Dark Triad construct requires a considerable amount of time and could induce participant fatigue (Webster & Jonason, 2013). Additionally, the different instruments employ different measurement techniques^1^, requiring scores on each measure to be standardized (Jonason, Li, Webster, & Schmitt, 2009). To address these limitations, two concise measures to assess the three components of the Dark Triad cluster in a single instrument have been developed and are now dominating Dark Triad research: The Dirty Dozen (Jonason & Webster, 2010) and the Short Dark Triad (SD3; Jones & Paulhus, 2014). In this article, we are focusing on the Dirty Dozen measurement scale.

The Dirty Dozen scale is composed of 12 items, four for each of the Dark Triad traits. While there is considerable support for the adequacy of the psychometric properties of this scale such as internal consistency, factor structure and, test-retest validity (e.g., Chiorri, Garofalo, & Velotti, 2019; Jonason et al., 2013; Jonason & Luévano, 2013; Jonason & McCain, 2012; Jonason & Webster, 2010; Jones & Paulhus, 2014), there are some concerns regarding the brevity of the instrument to the full-length measures of the Dark Triad traits. Some authors have proposed that this short measure may fail to capture some aspects of psychopathy and narcissism (e.g., Maples, Lamkin, & Miller, 2014; Miller et al., 2012). Another concern relates to the issue of whether this concise Dark Triad measurement instrument can capture the three separate traits or a bi-factor model where a narcissism factor and a combined Machiavellianism-psychopathy factor are found (e.g., Carter, Campbell, Muncer, & Carter, 2015; Egan, Chan, & Shorter, 2014). Various studies have used confirmatory factor analysis to explore the latent structure of the Dirty Dozen questionnaire and the best fit was found for a model where items load on both their dimension and a general Dark Triad factor (bi-factor model) (Jonason & Luévano, 2013; Jonason et al., 2013; McLarnon & Tarraf, 2017). To date, empirical evidence suggests that the Dirty Dozen is composed of three interrelated but distinct subscales. Jonason and Webster (2010) compared the three correlated factors Dirty Dozen model with a single, composite scale and with a hierarchical model in which three factors are nested into a higher-order factor. The study findings showed that the three-dimensional and hierarchical model of the Dirty Dozen were statistically equivalent and fit the data better than the one-dimensional model. In 2014, Jones and Paulhus created another short measure for the Dark Triad construct, the 27-item Short Dark Triad (SD3). When compared to the Dirty Dozen questionnaire, the SD3 retains a nomological network more similar to the original measures (i.e., NPI, Mach-IV, SRP; Jones & Paulhus, 2014; Maples, Lamkin, & Miller, 2014; Miller et al., 2017). However, the structure of the Dirty Dozen questionnaire appears to be more stable across different cultural contexts, which is crucial in the testing of measurement invariance, and therefore the scale seems to provide a reasonable tradeoff between efficiency and accuracy (Jonason & Luévano, 2013; Rogoza et al., 2020). Previous studies on gender differences among the Dark Triad traits have consistently found higher scores in men on all three dimensions, regardless of the measuring instrument used (Dark Triad measures or separate measures for each trait; see Barlett, 2016; Cale & Lilienfeld, 2002; Dowgwillo & Pincus, 2017; Furnham & Trickey, 2011; Grijalva et al., 2015; Jonason, Lyons, Bethell, & Ross, 2013; Muris et al., 2013; Muris et al., 2017; Paulhus & Williams, 2002; Pineda, Sandin, & Muris, 2020).

### Measurement invariance across gender in the Dirty Dozen Dark Triad version

Multigroup comparisons are only meaningful if it can be established whether or not components of the measurement model are equivalent or invariant across particular groups of interest, such as men and women (Byrne, 2012). The question of whether the three underlying theoretical constructs of the Dark Triad are being measured in the same way across gender is a matter of *measurement invariance*. Testing measurement invariance ensures that the observed indicators of the Dark Triad measure the same theoretical constructs (factors) across gender (Wang & Wang, 2020), thus possessing measurement equivalence. Establishing measurement invariance is a prerequisite for group comparison (Vandenberg & Lance, 2000). When measurement invariance is evidenced, it assures that (1) group comparisons are meaningful, (2) the same trait is measured across groups, and (3) group differences reflect true group differences. If measurement invariance assumptions do not hold, differences between groups cannot be interpreted unambiguously (Horn & McArdle, 1992). Thus, scores of men and women will not represent the underlying constructs of the Dark Triad equally, and observed group differences cannot be assumed to be accurate. So far, only a few studies have examined measurement invariance across gender for the Dirty Dozen Dark Triad measures. Klimstra et al. (2014) were the first to perform a rigorous measurement invariance test applied to the concise Dirty Dozen Dark Triad measure among two samples of Dutch-speaking adolescents in Belgium. Measurement invariance tests showed 1) strong invariance suggesting that the factor structure was similar for boys and girls in terms of the pattern of factor loadings and 2) strict invariance suggesting that the pattern of means across items was equivalent for boys and girls. Boys scored consistently higher than girls, especially on the psychopathy trait. Regarding Machiavellianism and narcissism, the evidence was somewhat less convincing, as boys scored higher on these traits in one sample, but not in the second sample. Chiorri et al. (2019) aimed to replicate the results of Klimstra et al. (2014) in a convenience sample of Italian adults (Study 1, 3, and 4) and undergraduate psychology students (Study 2) using an Italian translation of the Dirty Dozen and extending the replication study by assessing a larger range of invariance models. The results of the study revealed that the measurement model of the Dirty Dozen and its parameters were invariant across gender. Consistent with previous studies, they found that men scored higher on all Dark Triad traits than women for psychopathy and Machiavellianism, and to narcissism but to a lesser extent. Recently, Rogoza et al. (2020) conducted a test of measurement invariance of the Dirty Dozen across cultures using data from 49 countries. Support for full scalar invariance in men and women was established. Except for Asia, where no statistically significant differences in psychopathy across gender were found, in general, men scored significantly higher than women on all three traits. These findings suggest that the Dirty Dozen questionnaire can be used to provide reliable assessments of gender differences in Dark Triad traits.

### Overview of the present study and hypotheses

**Figure 1:**
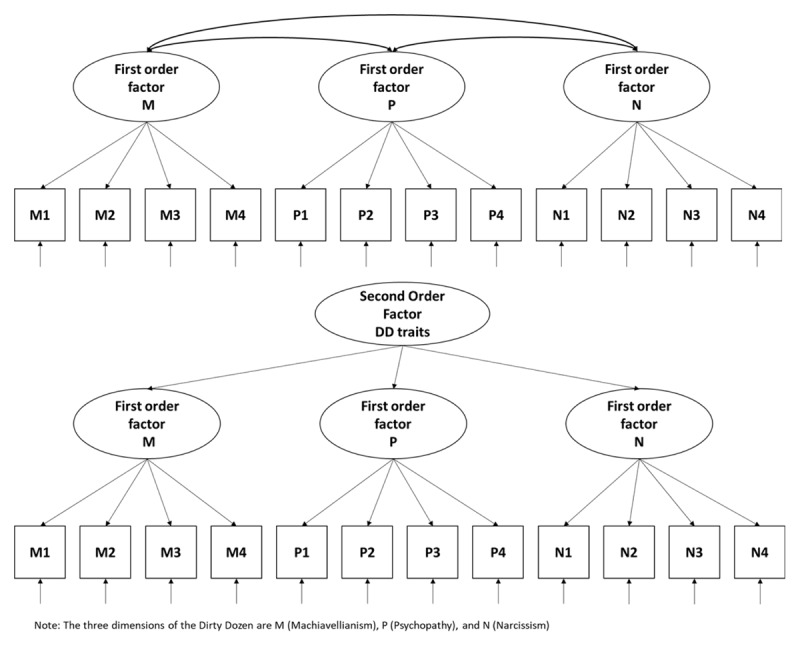
Above: Three dimensional model of the Dark Triad Dirty Dozen: three distinct but interrelated subdimensions. Below: Hierarchical model of the Dark Triad Dirty Dozen.

The main goal of the present study is to test for measurement invariance across gender for the Dutch version of the Dirty Dozen questionnaire in a large-scale adult community sample. In addition, (unique) relations between Dirty Dozen traits, low trait self-control, and acceptance of illegitimate norms are examined across gender. First, the three factors Dirty Dozen model is compared to a hierarchical second-order model (visualized in ***Figure 1***). Given findings from previous studies, we expect to find statistical equivalence between both models (Jonason & Webster, 2010; Schimmenti et al., 2019). Next, we hypothesize that the three correlated factors model would represent an adequate fit in both men and women (Hypothesis 1). We also hypothesize this three-factors model to be invariant across gender (Hypothesis 2). Furthermore, given the characterization of the Dirty Dozen traits as interpersonally aversive (Kurt & Paulhus, 2008), in particular callous social attitudes and impulsivity with implications for socially undesirable behaviors (Jonason et al., 2014), we conducted correlational analyses between Dark Triad personality traits and (1) low trait self-control and, (2) acceptance of illegitimate norms. These concepts were taken into account because, in contemporary theorizing about crime and delinquency, both low trait self-control and acceptance of illegitimate norms are among the strongest correlates of crime (e.g. Wikström, 2017, 2019). On the one hand, given that the three Dirty Dozen personality constructs share an antagonistic, dishonest and, malevolent core (Paulhus & Williams, 2002), we would expect to find positive correlations between the three Dirty Dozen traits and acceptance of illegitimate norms. Especially a strong correlation should be found with psychopathy that is typically considered to be the most nefarious (Hypothesis 3a). On the other hand, correlations with low trait self-control are less unequivocal. Theoretically, we would expect a *negative* correlation with Machiavellianism given that Machiavellian individuals are characterized by a strategic-calculating interpersonal orientation (Jones & Paulhus, 2014), whereas a *positive* correlation is expected with psychopathy given that disinhibition (related to impulsiveness) features in nearly all conceptions of psychopathy (Vize et al., 2018). Concerning narcissism, we expect to find a *positive* association given that lack of self-control is provided as an explanation for narcissists’ search for desired status and recognition (Vazire & Funder, 2006) (Hypothesis 3b).

## Method

### Participants and procedure

The study data were collected through a cross-sectional survey amongst a representative adult sample of people living in Belgium. Face-to-face interviews were conducted with citizens who were selected randomly, realized in cooperation with the municipality of Ghent. The participants were visited by trained interviewers, who were equipped with a laptop or tablet through which the respondents could access an online questionnaire hosted by Qualtrics. Part of the survey was filled out using a face-to-face interview method, but to address the sensitive nature of some of the questions, part of the survey was closed and only filled out by the participants, shielded from the interviewers. No incentives were given for participation. For a more detailed description of the study protocol (see Hardyns et al., 2019). The Institutional Review Board of Ghent University provided ethical approval for this study. The study sample was derived from a representative sampling technique. The sample was representative regarding age, gender (men versus women), and immigrant background (no immigrant background versus immigrant background). More than half of the participants (58.7% men; 60.4% women) reported having completed higher education (college or university). People with sufficient knowledge of the Dutch language and who did not reside in an institutional setting were deemed eligible for participation. In total, 1587 respondents (M_age_ = 48.06, *SD*_age_ = 18.64; 51.4% women) completed the questionnaire. Appendix 1 provides an overview of the sample descriptives.

### Measures

#### Dirty Dozen traits

The Dirty Dozen questionnaire consists of 12 statements aiming to capture three underlying traits: Machiavellianism, psychopathy, and narcissism. The Dirty Dozen short version was translated independently to Dutch by Dutch-speaking authors with very good knowledge of English. Some wordings of their translated version differed from the previously translated Dutch version that was examined by Klimstra et al. (2014) and Barelds (2016). However, this did not affect the content of the questionnaire. In line with the English original version, participants endorse 12 statements that are categorically scored on five levels, ranging from *do not agree at all* (1) to *completely agree* (5). Cronbach’s alphas were: Machiavellianism (*α* = 0.80) and narcissism (*α* = 0.80). Cronbach’s alpha for psychopathy (*α* = 0.64) did not surpass what is generally considered an acceptable Cronbach’s alpha cutoff (a Cronbach’s alpha >.70 is a widely used rule of thumb in social studies (Nunnally & Bernstein, 1994)).

#### Trait self-control

Trait self-control was measured with five items adapted from the self-control scale developed by Grasmick and colleagues (1993). Given that researchers have identified multidimensionality in the global self-control measure, our choice to retain the selected items builds on Steinberg’s dual systems model of neurobiological development that emphasizes two dimensions of low self-control: risk-seeking and impulsivity (Steinberg, 2010; Steinberg et al., 2008). In the present study, the following five items were used: “I often do things without thinking first”, “I have fun when I can, even if it gets me in trouble”, “Sometimes I will take a risk just for the fun of it”, “I say what I think, even if it’s not smart”, “I often immediately do what I feel like”. Items were scored on a 5-point Likert scale, ranging from “do not agree at all”(1) to “completely agree”(5). Responses were coded in such a way that high scores on the scale represented low trait self-control. Cronbach’s alpha of the scale was .69.

#### Acceptance of illegitimate norms

Acceptance of illegitimate norms was self-rated by participants with 4 items as previously used by Pauwels (2011) and Pauwels and Svensson (2013). Responses were given on a 5-point Likert scale ranging from “do not agree at all” (1) to “completely agree” (5). A sample item is “Rules are made to be broken”. Responses were coded in such a way that high scores on the scale represented a strong acceptance of illegitimate norms or poor personal morals. The scale reported a Cronbach’s alpha of .74.

### Analytic strategy

Firstly, measurement invariance is tested using multi-group first-order CFA for categorical variables (Bollen, 1989) in M*plus* version 7.11 (Muthén & Muthén, 2012). At each step of the procedure, a series of nested factor models, that place increasing restrictions on parameters across the two groups, are estimated. Before testing measurement invariance, a baseline model for each gender group is determined (Byrne, 2012). The hypothesized three-factor structure of the Dirty Dozen served as the initial model tested in the establishment of the baseline models for men and women separately. Evaluation of good model fit, i.e. a model that is consistent with the data, is assessed by using the following indices in combination: A non-significant *χ*^2^ is desired. However, *χ*^2^ statistic is highly sensitive to sample size. As such, the significance of the *χ*^2^ test should not be a reason by itself to reject a model (Wang & Wang, 2020); Comparative fit index (CFI) (Bentler, 1990) and Tucker Lewis Index (TLI; Tucker & Lewis, 1973) should be larger than .90, but values larger than .95 present better fit (Hu & Bentler, 1999)); Root mean square error of approximation (RMSEA), interpreted as: 0 = perfect fit; <.05 = close fit; .05–.08 = fair fit; .08–.10 = mediocre fit; and >.10 = poor fit (Byrne, 2012; Hu & Bentler, 1999; MacCallum et al., 1996). In addition, the 90% CI, computed for the RMSEA, is reported. Ideally, the lower value of the 90% CI should be very near-zero (or no worse than .05) and the upper value should be less than .08; Weighted Root Mean Squared Residual (WRMR), a residual-based model fit index. Perfect model fit is indicated by WRMR = 0 and increasingly higher values indicate worse fit (Kline, 2016). Although there is no absolute agreement on what constitutes a good fit, there is a consensus in these proposed criteria. Subsequently, measurement invariance testing is conducted. Invariance testing involves four different levels that form a nested hierarchy: configural invariance, weak factorial invariance, strong factorial, and strict factorial invariance (Kline, 2016; Meredith, 1993). Configural invariance requires that a measurement instrument measures the same common factors across groups. This implies that the patterns of item clusters in the configural model are identical across the groups. Once the baseline model is determined for men and women, these two models are combined into a multigroup model to form the configural model (Horn & McArdle, 1992) in which the same number of factors and the same pattern of factor loadings are specified in each group (Wang & Wang, 2020). This initial step in testing for configural invariance requires that no equality constraints are imposed on the parameters. The same parameters estimated in the baseline model for each gender group *separately* are again estimated in the configural model but now *simultaneously* (Byrne, 2006; Horn & McArdle, 1992; Reise et al., 1993; Vandenberg & Lance, 2000). Assuming the common factor model is configurally invariant across the groups, the next step in the process is testing weak factorial invariance. A weak factorial invariance model requires equivalence of the corresponding unstandardized factor loadings across groups (Kline, 2016). Factor loadings represent the strength of the linear relationships between the observed indicators and the underlying factors. Weak factorial invariance is tested by constraining the factor loadings to be equal (usually constrained to 0) and fitting the factor model to the sample data from each group simultaneously. If factor loadings are invariant across groups, then measures across groups are considered to be on the same scale (Wang & Wang, 2020) and common factors are deemed to have the same meanings across groups. Testing weak factorial invariance is the least restricted. If the weak factorial invariance hypothesis is supported, a more restricted model, a strong factorial model, is tested. Strong factorial invariance imposes equality constraints on all corresponding factor loadings and item thresholds and fits the model to the sample data from each group. A good model fit suggests that the model constraints are consistent with the data. Significant worsening of the fit suggests that the equal item thresholds hypothesis does not hold (Gregorich, 2006). Strict measurement invariance is the highest level to achieve. In this step of the modeling, the item factor loadings, item thresholds, and item residuals are held equal across the groups. However, if strong factorial invariance does not hold, no further invariance testing is necessary. Furthermore, many disciplines do not require item residual invariance, so that strict invariance is considered unnecessary (Bentler, 2006). Given that the twelve items of the Dirty Dozen are categorically scored on a five-point Likert scale and given that some items have piling of responses in the smallest or largest category, Weighted Least Squares Means and Variances (WLSMV) is utilized for model estimation (Kline, 2016). When WLSMV is used for model estimation and comparison, a two-stage approach, using the DIFFTEST-option in M*plus* is available for difference testing between the models (Asparouhov & Muthén, 2006; Muthén & Muthén, 2012). Additional fit criteria for model comparison include ΔCFI less than or equal to –.002 and ΔRMSEA larger than or equal to .007 (Meade et al., 2008). Secondly, bivariate correlations between trait self-control, acceptance of illegitimate norms, and Dirty Dozen traits are examined using SPSS Statistics 27. Secondly, we ran a series of SEM models in M*plus* Version 7.11 to examine the unique relations between the theoretical constructs. In the first series of models, low trait self-control is the exogenous variable, and Machiavellianism, psychopathy, and narcissism are the endogenous variables. In the second series of models, the three Dirty Dozen traits are the exogenous variables, and acceptance of illegitimate norms is the endogenous variable. SEM models are calculated across gender.

## Results

### Measurement invariance testing across gender

**Figure 2:**
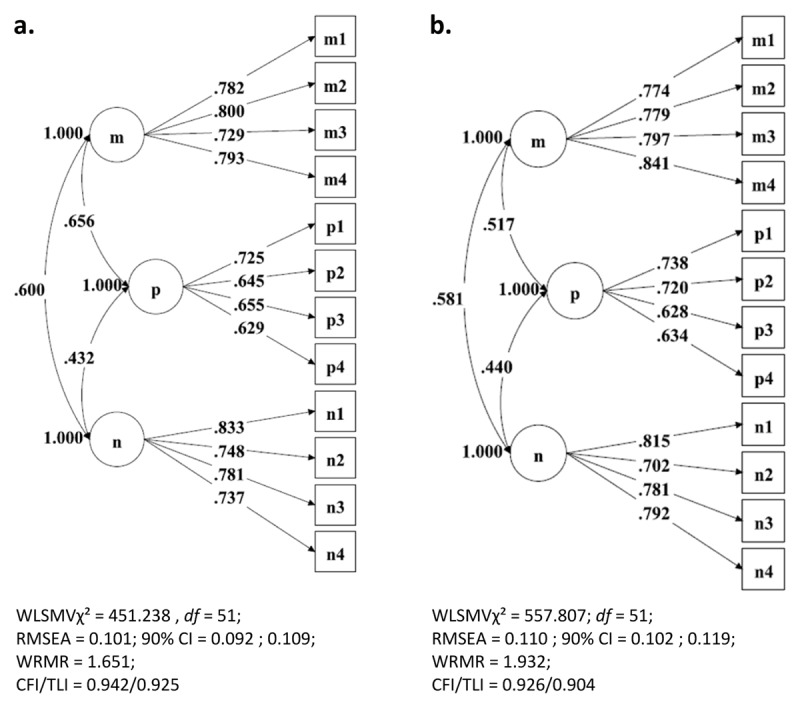
Separate hypothesized baseline models of Dirty Dozen factor structure for men and women. **a.** Separate hypothesized baseline models of Dirty Dozen structure for men (N = 772). **b.** Separate hypothesized baseline models of Dirty Dozen structure for women (N = 815).

Before invariance testing, we compared the three correlated factors model to a hierarchical model in which the three Dirty Dozen subdimensions are nested into a higher-order factor. Both models produced identical fits to the data. Further analyses were conducted using the three correlated factors model. Next, the baseline model across gender was determined (Byrne, 2012). Initial test results of the two baseline models for men and women, displayed in ***Figure 2a*** and ***2b***, show that the Dirty Dozen-12 items highly load to their underlying factors in the two samples.

***Table 1*** displays a summary of the model fit of the baseline model for women (N = 815) (WLSMV*χ*^2^ = 557.807, *df* = 51; RMSEA = .110; 90% CI = .102; .119; WRMR = 1.932; CFI/TLI = .926/.904) and for men: (N = 772) ((WLSMV*χ*^2^ = 451.238, *df* = 51; RMSEA = .101; 90% CI = .092; .109; WRMR = 1.651; CFI/TLI = .942/.925). Although RMSEA values indicate poor model fit, CFI/TLI values indicate good fit (>.90). We considered this three-factor model to best represent the hypothesized multigroup model under test. We did not conduct posthoc analyses, based on the modification indices. The pros and cons of post hoc analyses and post hoc model-fitting in SEM are largely debated in the literature. Because of the exploratory nature of these analyses, some scholars have severely criticized the practice (e.g. Cudeck & Browne, 1983). Other scholars have taken a more moderate stance on the matter (e.g. Byrne et al., 1989). Post hoc model fitting in SEM comes with the risk of capitalization on chance because model modification may be driven by characteristics of the sample on which the model was tested such as sample heterogeneity (MacCallum et al., 1992 in Byrne, 2012). No residual covariances between items were re-specified as freely estimated parameters. Turning to the evaluation of the configural model, WLSMV estimation of this model yielded the following goodness-of-fit statistics: WLSMV*χ*^2^ = 1013.266, *df* = 102; CFI/TLI = .934/.914; RMSEA = .106; 90% CI = .100 – .112; and WRMR = 2.542. Again, no post-host model fitting was allowed, so this configural model was taken as the baseline value against which the first comparison of nested models was made.

**Table 1:** Summary of Model Fit and *χ*^2^-Difference-Test statistics.


MODEL	WLSMV*χ*^2^	*df*	CFI/TLI	RMSEA	WRMR	DIFFTEST*χ*^2^ (Δ*df*) *p*	*ΔRMSEA*	*ΔCFI*

*Baseline (Women)*	557.807	51	.926/.904	.110 90%CI .110 – .119	1.932			

*Baseline (Men)*	451.238	51	.942/.925	.101 90%CI .092 – .109	1.651			

*Configural*	1013.266	102	.934/.914	.106 90%CI .100 – .112	2.542			

*Weak*	**899.294**	**111**	**.943/.932**	**.095** **90% CI** **.089 – .100**	**2.581**	**16.808** **(9)** ***p* < .052**	**.011**	*–*.**009**

*Strong*	1039.041	156	.936/.946	.084 90% CI .080 – .089	2.806	172.524 (45) *p* < .001	.011	.007


### Preferred model in bold

Fit statistics related to the weak factorial invariant model are: WLSMV*χ*^2^ = 899.294, *df* = 111; CFI/TLI = .943/.932; RMSEA = .095; 90% CI = .089; .100; and WRMR = 2.581. The null hypothesis of testing weak invariance that factor loadings are not significantly different across gender is retained. ΔWLSMV*χ*^2^ = 16.808, with 9 degrees of freedom and a probability of .052 suggests that factor loadings are not significantly different between men and women, cannot be rejected. By placing restrictions on the factor loadings, the model fit does not perform significantly worse than before. Although the *χ*^2^ DIFFTEST is marginally non-significant, with a Δ*CFI* = –.009 (change below the recommended threshold) and ΔRMSEA = .011 (change above the recommended threshold), we conclude that the Dirty Dozen measurement tool holds weak factorial equivalence across gender. Evidence of weak factorial invariance implies the same factor structure, the same cluster of items, and equal factor loadings across gender. We conclude that the underlying latent factors are measured the same way with the same metric in the two populations. Variances and covariances can be compared at the latent level (via SEM). Fit statistics related to the strong factorial invariant model are: WLSMV*χ*^2^ = 1039.041, *df* = 156; CFI/TLI = .936/.946; RMSEA = .084; 90% CI = .080 – .089; and WRMR = 2.806. The DIFFTEST-results are as follows: ΔWLSMV*χ*^2^ = 172.524, with 45 degrees of freedom and a probability of .001; Δ*CFI* = .007 and ΔRMSEA = .011. With a significant *χ*^2^ DIFFTEST and Δ*CFI* = .007 (change above recommended threshold), we conclude that the Dark Triad measurement tool does not hold strong measurement invariance across men and women. By placing constraints on the item thresholds, the model fit performs significantly worse than before. When strong measurement invariance is not evidenced, it means that item thresholds are not invariant across groups, suggesting that participants in at least one of the groups tend to respond systematically higher or lower to the items of the Dark Triad-scales, even if factor loadings are invariant across groups (Wang & Wang, 2020). Because strong factorial invariance was not evidenced in the Dirty Dozen measurement tool, the procedure for strict factorial testing was not performed. In sum, our findings suggest that factor loadings are invariant across gender. Recommended implications for attaining weak factorial invariance are that group comparisons are defensible concerning variances and covariances at the latent level (via SEM). Thus, we deemed it sensible to calculate relations between Dirty Dozen traits, low trait self-control, and acceptance of illegitimate norms, via SEM models, for men and women.

### Relations between Dirty Dozen traits, low trait self-control, and acceptance of illegitimate norms

***Table 2*** displays the bivariate correlations and SEM (standardized) regression coefficients, firstly for the three Dirty Dozen traits regressed on low trait self-control and secondly for the Acceptance of illegitimate norms regressed on the Dirty Dozen traits.

**Table 2:** Bivariate correlations and SEM regression coefficients for Dirty Dozen traits on trait self-control and Acceptance of illegitimate norms on Dirty Dozen traits (across gender).


	MACHIAVELLIANISM		PSYCHOPATHY		NARCISSISM	

**Men (*N* = 772)**						

	**r**	*β*	**r**	*β*	**r**	*β*

***Trait self-control***	.401***	.559***	.394***	.578***	.250***	.367***

**Women (*N* = 815)**						

	**r**	*β*	**r**	*β*	**r**	*β*

***Trait self-control***	.302***	.443***	.295***	.459***	.225***	.334***

**Acceptance of illegitimate norms**						

	**Men (*N* = 772)**			**Women (*N* = 815)**		

	**r**	*β*		**r**	*β*	

***Machiavellianism***	.400***	.340***		.291***	.255***	

***Psychopathy***	.420***	.398***		.412***	.498***	

***Narcissism***	.239***	*ns*		.167***	*ns*	


* p < .05 ** p < .01 *** p < .001 ns = not significant.

We found that Machiavellianism, psychopathy, and narcissism are positively associated with trait self-control in both subsamples. Across men, the strength of the associations between psychopathy and trait self-control and between Machiavellianism and trait self-control is approximately the same, resp. *r* = .394, *β* = .578; *p < .001* and *r* = .401, *β* = .559; *p < .001*, followed by narcissism (*r* = .250, *β* = .367; *p < .001*). That is, men with higher reported levels of Machiavellianism, psychopathy, and narcissism also report higher levels of low trait self-control. The same patterns are found in women, although the coefficients are slightly lower: psychopathy and trait self-control (*r* = .295, *β* = .459; *p < .001*), Machiavellianism and trait self-control (*r* = .302, *β* = .443; *p < .001*) followed by narcissism and trait self-control (*r* = .225, *β* = .334; *p < .001*). Turning to the associations between Dirty Dozen traits and acceptance of illegitimate norms, it can be seen that the latter is positively associated with both Machiavellianism and psychopathy in both subsamples. In the subgroup of men, the strength of the associations is approximately the same, resp. Machiavellianism: *r* = .400, *β* = .340; *p < .001* and for psychopathy: *r* = .420, *β* = .398; *p < .001*. That is, men who reported higher levels of Machiavellianism and psychopathy also reported higher acceptance of illegitimate norms. The strength of both associations can be interpreted as weak-moderate (for an overview of three commonly used interpretations of the *r* values, see Akoglu, 2018). In the subgroup of women, the pattern is more pronounced. The association between psychopathy and acceptance of illegitimate norms is moderate-strong (*r* = .412, *β* = .498; *p < .001*) followed by a weak association with Machiavellianism (*r* = .291, *β* = .255; *p < .001*). No unique correlations were found with narcissism.

## Discussion

The present study assessed measurement invariance across gender of the Dutch version of the Dark Triad Dirty Dozen measure among a representative adult sample in Belgium (*N* = 1587). The main conclusions were the following. Firstly, we found good internal consistency values in terms of Cronbach’s alpha for the subscales Machiavellianism and psychopathy but not for narcissism. This is consistent with previous studies using the Dirty Dozen measure (Chiorri et al., 2019; Jonason & Tost, 2010; Klimstra et al., 2014; Pechorro et al., 2019). Secondly, we expected to find measurement models, across gender, that fit the data well for a three-factor structure (Hypothesis 1). Our findings suggest that the three correlated factor structure of the Dutch Dirty Dozen measure represents only a poor fit to the data. Thirdly, in line with previous studies, we expected to find the factor structure to be similar for men and women in terms of the pattern of factor loadings (weak factorial invariance) and the pattern of items thresholds (strong factorial invariance) (Hypothesis 2). Hypothesis two was tested using a multigroup confirmatory analysis. The results provided evidence for weak factorial invariance only across adult men and women. Thus, the second hypothesis was not corroborated. This result could be due to the use of a representative heterogeneous sample. In contrast, other studies, using more homogeneous convenience samples, found evidence for strict measurement invariance of the Dirty Dozen measure across gender in high school students (Klimstra et al., 2014), in an adult community sample (Chiorri et al., 2019), among a sample of at-risk Portuguese youths (Pechorro et al., 2019) and across cultures in eight world regions (using convenience samples of university students) (Rogoza et al., 2020). Achieving weak factorial invariance across gender allowed further comparison of covariances on the latent level. Subsequently, associations between the three Dirty Dozen traits, low trait self-control, and personal morals were explored across gender. In addition, a series of SEM models were run to identify unique associations (Hypothesis 3a & 3b). Contrary to our hypotheses, all three dimensions of the Dirty Dozen showed significant positive relations with low trait self-control. Pearson’s correlation coefficients were highest in the association with Machiavellianism and psychopathy for men as compared to women. To date, empirical studies examining the link between Dark Triad personality traits and low trait self-control found mixed results (e.g. Barelds, 2016; Jones & Paulhus, 2011; Miller et al., 2009; Pechorro et al., 2019; Vize et al., 2018). One explanation could be that different measures of self-control are used and/or data come from multiple different samples. Self-control ability is a complex and multidimensional construct that is used (1) in a specific sense to denote the capacity to resist temptation but (2) also in a broader sense to refer to effective self-regulation (Nigg, 2017). Confounding the concept with impulsivity, risk-seeking, disinhibition impedes comparison of empirical findings across different studies. Furthermore, the present study observed positive moderate associations between Machiavellianism, psychopathy and reported acceptance of illegitimate norms for both men and women (but no unique associations with narcissism). This result is consistent with the notion that all three Dark Triad traits share a common callous core that is considered maladaptive in social interactions (Jones & Figueredo, 2013), although narcissism is generally considered the least socially aversive trait of the cluster (Pailing et al., 2014). Other studies found that Dark Triad traits are positively correlated with scores on Aggression Questionnaire (e.g. Chiorri et al., 2019; Jonason & Webster, 2010). Pechorro et al. (2019) found evidence for mostly similar positive associations between the three dimensions of the Dirty Dozen with scores on a self-reported delinquency scale, with narcissism showing slightly lower values. Given that our analyses were exploratory, future research could make a more formal comparison between the two subpopulations by testing for interaction. For example, associations between Dirty Dozen traits, low trait self-control, and personal morals could be tested simultaneously by using the method of multiple groups comparison (via SEM) (Kline, 2016). The strength of the present study is that it is one of the first to test for measurement invariance of the Dutch version of the Dirty Dozen across gender in a representative adult sample in Belgium. Though there are some limitations to address. First, it is necessary to mention that, although we used a representative sampling technique (see paragraph Method), more than half of the participants reported a higher education level (college or university). Higher educated groups (e.g. students) are often overrepresented in social surveys. The cognitive burden incurred in answering and comprehending survey questions might result in higher survey cooperation by the higher educated (Stoop, 2012). In addition, the present study was limited by the fact that all findings are based on self-reported data. Also, as the scale evaluates self-perceived “dark” personality traits, responses could be contaminated by social desirability, especially considering the Dark Triad traits are characterized by tendencies towards self-promotion (Paulhus & Williams, 2002). However, previous research has demonstrated that self-report measurements of the Dark Triad can be quite accurate (Jonason & Webster, 2010), and a recent study by Kowalski et al. (2018) found that only narcissism is associated with social desirability. Another important shortcoming is related to the external validity of the measurement instrument. In our operationalization of the Dark Triad personality traits, we used a concise measure. While there is considerable support for the adequacy of the psychometric properties of this scale (e.g., Chiorri, Garofalo, & Velotti, 2019; Jonason et al., 2013; Jonason & Luévano, 2013; Jonason & McCain, 2012; Jonason & Webster, 2010; Jones & Paulhus, 2014), there are some concerns regarding the brevity of the instrument to full-length measures of the Dark Triad traits (Lee et al., 2013; Miller et al., 2012). Some authors have proposed that this short measure may fail to capture some aspects of psychopathy and narcissism (e.g., Maples, Lamkin, & Miller, 2014). In addition, Cronbach’s alpha for psychopathy did not surpass the rule of thumb threshold of .70 which suggests questionable internal reliability. Schmitt (1996) takes a more liberal stance by suggesting that measurement instruments with quite low values of alpha can still prove useful and that no general threshold, such as .70, exists where alpha becomes acceptable. In addition, alternative measures of reliability exist (e.g. Hayes & Coutts, 2020; Revelle & Zinbarg, 2009). Cronbach’s alpha is, in part, influenced by the number of items in a scale. Given that the three-factor measures of the Dark Triad Dirty Dozen are composed of only four items each, it is likely to have relatively lower levels of internal consistency (Jonason & Webster, 2010).

To conclude, since we were not able to achieve full measurement invariance across gender of the Dutch Dirty Dozen measure, observed group differences should be interpreted with caution. Jones & Paulhus (2014) stated that the empirical literature does not favor the use of the Dirty Dozen measure given its extreme brevity that has drawn criticism. An alternative 27-items measure of the Dark Triad has been developed, the Short Dark Triad (SD3) (Jones & Paulhus, 2014). Maples et al. (2014) examined and compared validity scores on both the Dirty Dozen and the Short Dark Triad. In their conclusion, the authors note that in cases where time is of the essence and a short measure of the Dark Triad is required, the SD3 scales yield effect sizes that are more consistent with the underlying constructs as they are measured using more established and validated Dark Triad scales. In Belgium, measurement invariance of the SD3 is previously tested in a cross-cultural study, however, the French translation of the measure was used (Atitsogbe et al., 2020). Future research should examine the psychometric properties of the SD3 Dutch version as a short alternative self-report measure.

## Additional Files

The additional files for this article can be found as follows:

10.5334/pb.1106.s1 Appendix 1. Demographic Composition of Study Sample.

10.5334/pb.1106.s2 Appendix 2. Scale Items, Factor Loadings, and Reliability Analysis for Dark Triad Traits, acceptance of illegitimate norms and trait self-control.

10.5334/pb.1106.s3 Appendix 3. Descriptive statistics of theoretical constructs (Full sample – *N* = 1587).

## Footnotes

The Narcissistic Personality Inventory (NPI) consists of 40 items that are answered in a forced-choice dichotomous scale while the Self-Report of Psychopathy Scale and the Mach-IV are generally scored on a 5-point Likert scale.
