# Supplementary material for: Testing Measurement Invariance of the Dark Triad Dirty Dozen in a Belgian Adult Sample
Source: Psychol Belg. 2021 Dec 22;61(1):377–90. doi: 10.5334/pb.1106 (PMC8698221; doi:10.5334/pb.1106)
Supplement: Appendix 1. — Demographic Composition of Study Sample. [file pb-61-1-1106-s1.pdf]

APPENDIX

Appendix 1

Demographic Composition of Study Sample

	**Total sample (*n* = 1587)**	**Women (*n* = 815)**	**Men (*n* = 772)**
Mean age in years (SD)	48.06 (18.64)	49.16 (18.87)	46.91 (18.33)
N per age category (%)			
18-24	158 (10.0%)	76 (9.3%)	82 (10.6%)
25-34	318 (20.0%)	156 (19.1%)	162 (21.0%)
35-44	278 (17.5%)	139 (17.1%)	139 (18.0%)
45-54	232 (14.6%)	122 (15.0%)	110 (14.2%)
55-64	243 (15.3%)	127 (15.6%)	116 (15.0%)
65+	358 (22.6%)	195 (23.9%)	163 (21.1%)
N per background (%)			
No immigrant background	1409 (88.8%)	736 (90.3%)	673 (87.2%)
Immigrant background	178 (11.2%)	79 (9.7%)	99 (12.8%)
N per Education/Qualification (%)			
No qualification	53 (3.3%)	30 (3.7%)	23 (3 %)
Lower secondary school	116 (7.3%)	65 (8 %)	51 (6.6%)
Higher secondary school	473 (29.8%)	228 (28%)	245 (31.7%)
Higher education (college or university)	945 (59.5%)	492 (60.4%)	453 (58.7%)
