# Supplementary material for: Testing Measurement Invariance of the Dark Triad Dirty Dozen in a Belgian Adult Sample
Source: Psychol Belg. 2021 Dec 22;61(1):377–90. doi: 10.5334/pb.1106 (PMC8698221; doi:10.5334/pb.1106)
Supplement: Appendix 2. — Scale Items, Factor Loadings, and Reliability Analysis for Dark Triad Traits, acceptance of illegitimate norms and trait self-control. [file pb-61-1-1106-s2.pdf]

Appendix 2

Scale Items, Factor Loadings, and Reliability Analysis for Dark Triad Traits, acceptance of illegitimate norms and trait self-control

Scale construct & items	Mean	*SD*	Factor loadings λ	Cronbach's α
**Dark Triad – Machiavellianism**				.80
I tend to manipulate others to get my way	1.82	0.953	.786	
I have used deceit or lied to get my way	2.18	1.123	.794	
I have use flattery to get my way	2.42	1.204	.762	
I tend to exploit others towards my own end	1.61	0.822	.819	
**Dark Triad – Psychopathy**				.64
I tend to lack remorse	1.66	0.961	.729	
I tend to be unconcerned with the morality of my actions	1.62	0.929	.681	
I tend to be callous or insensitive	1.61	0.902	.674	
I tend to be cynical	2.28	1.162	.653	
**Dark Triad – Narcissism**				.80
I tend to want others to admire me	2.52	1.149	.823	
I tend to want others to pay attention to me	2.82	1.142	.706	
I tend to seek prestige or status	2.23	1.086	.787	
I tend to expect special favors from others	1.74	0.871	.769	
**Acceptance of illegitimate norms**				.74
Rules are made to be broken	1.94	0.980	.705	
If honest ways to achieve something fail, then use the dishonest ways	1.64	0.897	.750	
It is okay to break the rules when you are provoked	1.76	0.918	.828	
It is okay to fight when you are provoked	1.52	0.912	.625	
**Trait Self-control**				.69
I often do things without thinking first	2.24	1.106	.518	
I have fun when I can, even if it gets me in trouble	2.38	1.086	.639	
Sometimes I will take a risk just for the fun of it	2.09	1.113	.655	
I say what I think, even if it's not smart	2.69	1.209	.569	
I often immediately do what I feel like	2.63	1.114	.645	
