# Supplementary material for: Testing Measurement Invariance of the Dark Triad Dirty Dozen in a Belgian Adult Sample
Source: Psychol Belg. 2021 Dec 22;61(1):377–90. doi: 10.5334/pb.1106 (PMC8698221; doi:10.5334/pb.1106)
Supplement: Appendix 3. — Descriptive statistics of theoretical constructs (Full sample – N = 1587). [file pb-61-1-1106-s3.pdf]

Appendix 3

Descriptive statistics of theoretical constructs (Full sample – *N*= 1587)

	Mean	*SD*	Min	Max	Skew	SE
**Machiavellianism**	2.01	0.82	1	4.25	0.52	0.06
**Psychopathy**	1..79	0.69	1	5.00	0.93	0.06
**Narcissism**	2.33	0.84	1	5.00	0.15	0.06
**Low trait self-control**	2.41	0.75	1	5.00	.037	0.06
**Acceptance of illegitimate norms**	1.71	0.69	1	5.00	1.19	0.06

Descriptive statistics of theoretical constructs (Men – *N*= 772)

	Mean	*SD*	Min	Max	Skew	SE
**Machiavellianism**	2.15	0.84	1	4.25	0.35	0.09
**Psychopathy**	1.96	0.72	1	5.00	0.73	0.09
**Narcissism**	2.41	0.85	1	5.00	0.05	0.09
**Low trait self-control**	2.45	0.76	1	5.00	0.34	0.09
**Acceptance of illegitimate norms**	1.81	0.71	1	5.00	0.99	0.09

Descriptive statistics of theoretical constructs (Women – *N*= 815)

	Mean	*SD*	Min	Max	Skew	SE
**Machiavellianism**	1.88	0.78	1	4.25	0.69	0.09
**Psychopathy**	1.64	0.62	1	5.00	1.14	0.09
**Narcissism**	2.25	0.83	1	5.00	0.25	0.09
**Low trait self-control**	2.37	0.75	1	5.00	0.39	0.09
**Acceptance of illegitimate norms**	1.62	0.66	1	5.00	1.43	0.09
